# A Comparative Study of Methods for Detecting Extraterrestrial Life in Exploration Missions to Mars and the Solar System II: Targeted Characteristics, Detection Techniques, and Their Combination for Survey, Detection, and Analysis

**DOI:** 10.1089/ast.2022.0148

**Published:** 2023-10-17

**Authors:** Keigo Enya, Akihiko Yamagishi, Kensei Kobayashi, Yoshitaka Yoshimura, Elizabeth J. Tasker

**Affiliations:** ^1^ Department of Solar System Sciences, Institute of Space and Astronautical Science, Japan Aerospace Exploration Agency, Sagamihara, Japan.; ^2^ Space and Astronautical Science, Graduate Institute for Advanced Studies, SOKENDAI, Hayama, Japan.; ^3^ Department of Applied Life Science, School of Life Science, Tokyo University of Pharmacy and Life Sciences, Tokyo, Japan.; ^4^ Department of Chemistry, Yokohama National University, Yokohama, Japan.; ^5^ Department of Earth and Planetary Science, Tokyo Institute of Technology, Tokyo, Japan.; ^6^ Department of Life Science, Tamagawa University, Machida, Japan.

**Keywords:** Extraterrestrial life, Life detection, Life detection method, Life detection instruments, Mars, Solar system

## Abstract

We present a comparative study of the methods used in the search for extraterrestrial microorganism life, including a summary table where different life-detection techniques can be easily compared as an aid to mission and instrument design aimed at life detection. This is an extension of previous study, where detection techniques for a series of target characteristics and molecules that could constitute a positive life detection were evaluated. This comparison has been extended with a particular consideration to sources of false positives, the causes of negative detection, the results of detection techniques when presented regarding terrestrial life, and additional science objectives that could be achieved outside the primary aim of detecting life. These additions address both the scientific and programmatic side of exploration mission design, where a successful proposal must demonstrate probable outcomes and be able to return valuable results even if no life is found. The applicability of the life detection techniques is considered for Earth life, Earth-independent life (life emerging independently from that on Earth,) and Earth-kin life (sharing a common ancestor with life on Earth), and techniques effective in detecting Earth life should also be useful in the detection of Earth-kin life. However, their applicability is not guaranteed for Earth-independent life. As found in our previous study, there exists no realistic single detection method that can conclusively determine the discovery of extraterrestrial life, and no method is superior to all others. In this study, we further consider combinations of detection techniques and identify imaging as a valuable addition to molecule detection methods, even in cases where there is insufficient resolution to observe the detailed morphology of a microbial cell. The search for extraterrestrial life is further divided into a survey-and-detection and analysis-and-conclusion step. These steps benefit from different detection techniques, but imaging is necessary for both parts.

## 1. Introduction

The search for extraterrestrial life is one of the most important goals for space science in the near future. Missions to search for evidence of extraterrestrial life on the planets and small bodies of our Solar System feature prominently in the US National Science Foundation Planetary Decadal Survey and the European Space Agency's “Voyage 2050” themes that will guide the selection of some of the largest space missions through the coming decades.

The first mission to search for extraterrestrial life on the surface of another planet was the NASA Viking mission in the 1970s. Viking performed multiple experiments with the goal of detecting life on Mars, which included (1) the thermal volatilization gas chromatography/mass spectrometry (TV-GC/MS) experiment (Biemann *et al*., 1977), (2) the gas exchange (GEX) experiment (Oyama and Berdahl, 1977), (3) the labeled release (LR) experiment (Margulis *et al*., 1979), and (4) the pyrolytic release (PR) experiment (Horowitz *et al*., 1977). These experiments were historic in the context of astrobiology, but the interpretation of the data remains controversial. As a result, Viking was not able to conclusively detect life on Mars. The Mars missions that have been launched since Viking have had science objectives related to astrobiology with a focus primarily on materials and the environments related to life, rather than the detection of extant life.

Searching for biosignatures through high-precision observations of extrasolar planets is another promising approach to detecting life elsewhere in the galaxy (*e.g*., Fujii *et al.*, 2018). However, this method cannot provide direct images of life-forms. The Search for Extraterrestrial Intelligence (SETI) is also an essential component in the search for life but must overlook nonintelligent life (*e.g.*, Drake and Sobel, 1992; Tarter, 2004). These approaches are, therefore, complementary to the exploration of our own solar system.

In the history of life on Earth, large multicellular life evolved from smaller microorganisms. It is difficult to believe that multicellular life can emerge in the absence of smaller microorganisms anywhere in the universe. It can, therefore, be supposed that there exist environments inhabited only by microorganisms, but none in which only large multicellular organisms exist. Should life exist beyond Earth, microorganisms will inevitably be present. The search for this most common form of life can be performed through *in situ* exploration.

However, the design of a mission or instrument to detect unknown forms of microorganisms is far from trivial. We, therefore, started by considering the characteristics that can be targeted in the search for terrestrial life. Characteristics of life on Earth that are believed to be fundamental were first selected, and the techniques required to identify their presence were identified. Issues in sensitivity and instrument weight must also be carefully considered during the planning of any mission.

These issues were previously reported in a comparative study published by this group with the goal of detecting extraterrestrial microorganisms in an exploration mission to Mars and other locations in the Solar System (Enya *et al*., 2022a, hereafter Paper-I). This previous study selected characteristics and molecules that could be detected and analyzed to indicate the presence of extraterrestrial life and evaluated the search techniques for each. The main focus in Paper-I was a comparative table that summarized the characteristics and detection methods to act as a reference in extraterrestrial life detection experiments.

In the present article, the comparison is expanded to consider a number of important issues that must be considered in mission design and selection. These include the importance of false positives both for correctly identifying life and for maximizing scientific gain in exploration missions where the presence of the sought target is unknown, as well as a fuller consideration of the results for performing the detection techniques on terrestrial organisms. In the Discussion, the major conclusions of Paper-I are then re-evaluated based on the extended comparison and found to be overall unchanged. The most effective combination of life detection methods is addressed and highlights the importance of imaging in any instrument suite. Finally, the steps in an extraterrestrial life exploration mission are divided into two main parts that favor the strengths of different detection methods and can be used as a guide to ensure the mission is worthwhile even if life is not discovered. The studies included in this work primarily suppose Mars as a target; however, they are valid for a wide variety of celestial bodies in the Solar System.

## 2. Targeted Characteristics and Detection Techniques

The targeted characteristics for identifying extraterrestrial microorganisms and the techniques that can be employed in detecting each specific characteristic are presented in Table 1. This tabular summary is based on the table developed in Paper-I, which primarily focused on the sensitivity of different detection techniques for each characteristic. This study extends the table to a more thorough consideration of the detection results when applied to terrestrial microbes (our only current test bed), the sources of false positives, and potential scientific achievements in the absence of a life detection. As explored in the discussion, these additions are aimed at providing a more multifaceted view of each detection technique that can be used not only to develop instruments for studies of extraterrestrial life but also to maximize the use of the expected return data. As with Paper-1, the martian surface is used as the primary site for investigating the existence of microorganisms, but similar principals can also be applied to other sites of interest within the solar system, such as the icy moons and small bodies.

**Table 1.** Summary of the Targeted Characteristics and Detection Techniques Used for Extraterrestrial Microorganism Search

	(A)	(B)	(C)	(D)	(E)	(F)	(G)	(H)	(I)	(J)	(K) Generality for terrestrial microorganism					(L) Source of false positive (except Earth contamination)		(M)	(N)	(O)	(P)
											(Ka)	(Kb)	(Kc)	(Kd)	(Ke)	(La)	(Lb)				
	Targeted characteristics (major classification)	Targeted characteristics (details)	Content in a cell	Technique	Technique (details)	Special pretreatment requiring human operation in ground-based experiments	Instrument mass (ground-based)	Sensitivity for targeted characteristics	Observation volume or mass	Sensitivity (cell density)	Probability of having targeted characteristics	Detection success rate	Essential difficulty	Detectability for living and dead, and their distinguishability (if both are detectable)	Case of detection after long-term	Possibility of targeted characteristics generated by nonlife	Possibility of detecting nontargeted characteristics	Universality (considered) of targeted characteristics among Earth-independent microorganisms	Instrument for space mission	Possible meaning of negative detection (with the exception of “no life” and “life exists but under the detection limit of this method”)	Scientific objectives other than life search
(1)	Cell proliferation			Liquid culture	Sample acquisition; mixing with media; incubation; turbidity measurement or flow cytometry	—	50 kg	1 Cell	Arbitrary	1 Cell/sample	(1) 100%	0–1%	The determination of proliferation conditions is difficult	Living	Freeze-dried cells (in laboratory), dried cell pellets surviving in the space vacuum in the dark (confirmed: 3 years, expected: tens of years), hyper-halophile cells from the liquid inclusion in crystal halite	No	Crystallization or precipitation of inorganic materials	High probability of having proliferation capability	—	Just culture conditions not suitable, dead organisms only	—
(2)				Solid culture	Sample acquisition; sample spreading on a medium; incubation; colony counting	—	2 kg	1 Cell	Arbitrary	1 Cell/sample	(2) 100%	0–1%	The determination of proliferation conditions is difficult	Living	—	No	No	High probability having proliferation capability	—	Just culture conditions not suitable, dead organisms only	—
(3)	Cell morphology^a^		(1 μm diameter)	Optical microscopy	Sample acquisition; sample preparation; (staining); observation using an optical microscope	For transmissive observation of rock-like sample, slicing; pasting onto a slide glass; polishing	20 kg	1 Cell	10 × 10 × 0.05 mm	1.3 × 10^3^ cells/g of soil	(3) 100%	100%	—	Living, dead	Oldest cell fossils (3.5 billion-year-old rocks)	—	Abiotic organic globules, inorganic globules	High probability of having a cellular structure	—	Irregular morphology (solo or aggregates) not identified as organisms, hidden organisms (*e.g*., in crack of mineral particle), organisms not stained by the pigment used	Imaging of aggregates of abiotic organic compounds and inorganic particles
(4)				Fluorescence microscopy	Sample acquisition; staining; observation using a fluorescence microscope	—	20 kg	1 Cell	10 × 10 × 0.05 mm	1.3 × 10^3^ cells/g of soil	(4) 100%	100%	—	Living, dead, distinction^b^	—	—	Abiotic organic globules, inorganic globules	High probability of having a cellular structure containing organic molecules	LDM	Irregular morphology (solo or aggregates) not identified as organisms, hidden organisms (*e.g*., in crack of mineral particle), organisms not stained by the pigment used	Imaging of aggregates of abiotic organic compounds and inorganic particles
(5)				Raman microscopy	Sample acquisition; observation using a Raman microscope	—	50 kg	1 Cell	10 × 10 × 0.05 mm	1.3 × 10^3^ cells/g of soil	(5) 100%	100%	—	Living, dead	—	—	Abiotic organic globules, inorganic globules	High probability of having a cellular structure containing organic molecules	RLS, SHERLOCK	Irregular morphology (solo or aggregates) not identified as organisms, hidden organisms (*e.g*., in crack of mineral particle), organisms not having the particular Raman signal used	Imaging of aggregates of abiotic organic compounds and inorganic particles
(6)				UV fluorescence microscopy	Sample acquisition; observation using a UV fluorescence microscope	—	20 kg	1 Cell	10 × 10 × 0.05 mm	1.3 × 10^3^ cells/g of soil	(6) 100%	100%	—	Living, dead	—	—	Abiotic organic globules, inorganic globules	High probability of having a cellular structure containing organic molecules	SHERLOCK	Irregular morphology (solo or aggregates) not identified as organisms, hidden organisms (*e.g*., in crack of mineral particle), organisms not having the particular fluorescence signal used	Imaging of aggregates of abiotic organic compounds and inorganic particles
(7)				Transmission electron microscopy	Sample acquisition; sample preparation; staining; observation using a transmission electron microscope	Ultrathin slicing, negative staining, or shadowing	100 kg	1 Cell	200 × 200 × 15 μm	1.1 × 10^5^ cells/g of soil	(7) 100%	100%	—	Living, dead	Extracellular proteins of mammoth (million-year-old)	—	Abiotic organic globules, inorganic globules	High probability of having a cellular structure	—	Irregular morphology (solo or aggregates) not identified as organisms, hidden organisms (*e.g*., in crack of mineral particle)	Imaging of organic and inorganic particles with high resolution (though they cannot be distinguished)
(30)		Enzyme		Catalytic activity assay	Sample acquisition; cell destruction; water extraction; substrate addition; UV-VIS or fluorescence	Cell destruction (mechanical, freeze and thaw, *etc.*)	10 kg	1 μg/mL	0.1 g	10^6^ cells/g of soil	(30) 100%	100%	—	Living, very fresh dead	—	No	Abiotic catalytic organic compounds, abiotic catalytic inorganics	High probability of having some catalytic activities	—	Possibility of organisms that do not use enzyme, reaction conditions not suitable	Abiotic catalytic organic compounds, abiotic catalytic inorganics
(31)				Fluorescence microscopy	Sample acquisition; staining; observation using a fluorescence microscope	—	20 kg	1 Cell	10 × 10 × 0.05 mm	1.3 × 10^3^ cells/g of soil	(31) 100%	100%	—	Living, very fresh dead	—	No	Abiotic catalytic organic globules, abiotic catalytic inorganic globules	High probability of having some catalytic activities	LDM	Enzyme is present but diffused, possibility of organisms that do not use enzyme, reaction conditions not suitable	Abiotic catalytic organic globules, abiotic catalytic inorganic globules
(20)				GC/MS	Sample acquisition; cell destruction; water extraction; hydrolysis; derivatization; GC/MS	Cell destruction (mechanical, freeze and thaw, *etc.*)	50 kg	10 fmol (100 cell)	0.1 g	10^3^ cells/g of soil	(20) 100%	100%	—	Living, dead	Amino acids in Murchison meteorite (4.6-billion-year-old or older), collagen from *Tyrannosaurus rex* fossil bones (68 million-year-old)	Abiotic amino acids	No	High probability of having a cellular structure containing polymers of amino acids	SAM	Quite severe environment where amino acids are destroyed	Abiotic amino acids
(21)				LDI-MS	Sample acquisition; laser desorption; MS	—	100 kg	1.2 fmol/mm^2^ (when Trp is targeted)	0.2 mm diameter × 0.05 mm	8 × 10^4^ cells/g of soil (when Trp is targeted)	(21) 100%	100%	—	Living, dead	—	Abiotic amino acids	Compounds with similar molecular weight	High probability of having a cellular structure containing polymers of amino acids	ORIGIN	Quite severe environment where amino acids are destroyed	Abiotic amino acids
(22)				Fluorescence microscopy	Sample acquisition; staining; observation using a fluorescence microscope	—	20 kg	1 Cell	10 × 10 × 0.05 mm	1.3 × 10^3^ cells/g of soil	(22) 100%	100%	—	Living, dead	—	Abiotic amino acids	Abiotic organic globules, inorganic globules	High probability of having a cellular structure containing polymers of amino acids	LDM	Amino acids existing but diffused, quite severe environment where amino acids are destroyed	Aggregates of abiotic amino acids
(23)		Nucleic acid	0.1 pg	UV absorption	Sample acquisition; cell destruction; partial purification; UV absorption measurement at 260 nm	Cell destruction (mechanical, freeze and thaw, *etc.*); partial purification (phase separation or column chromatography)	0.3–5 kg	1 μg/mL	1 mL	10^7^ cells/g of soil	(23) 100%	100%	—	Living, dead	—	Never been found in natural environments; however, experiments suggest nucleic acids could be synthesized in natural environments	Compounds with 260 nm absorption (can be identified to some extent by precise spectrum analysis)	Not sure if the cells contain nucleic acid as genetic material	—	Possibility of non-DNA/RNA-type organisms	Abiotic nucleic acids
(24)				Fluorescence microscopy	Sample acquisition; staining; observation using a fluorescence microscope	—	20 kg	1 Cell	10 × 10 × 0.05 mm	1.3 × 10^3^ cells/g of soil	(24) 100%	100%	—	Living, dead, distinction^c^	—	Never been found in natural environments; however, experiments suggest nucleic acids could be synthesized in natural environments	Abiotic organic globules, inorganic globules	Not sure if the cells contain nucleic acid as genetic material	LDM	Nucleic acids existing but diffused, possibility of non-DNA/RNA-type organisms	Aggregates of abiotic nucleic acids
(25)		DNA	0.01 pg	UV absorption	Sample acquisition; cell destruction; partial purification; UV absorption measurement at 260 nm	Cell destruction (mechanical, freeze and thaw, *etc.*); partial purification (phase separation or column chromatography)	0.3‒5 kg	1 μg/mL	1 mL	10^7^ cells/g of soil	(25) 100%	100%	—	Living, dead	—	No	Compounds with 260 nm absorption (can be identified to some extent by precise spectrum analysis)	Not sure if the cells contain DNA as genetic material	—	Possibility of non-DNA-type organisms	—
(26)				DNA sequencing	Sample acquisition; cell destruction; purification; DNA sequencing	Cell destruction (mechanical, freeze and thaw, *etc.*); purification (phase separation or column chromatography)	0.3‒5 kg	1 Molecule	Not applicable	10^7^ cells/g of soil	(26) 100%	100%	—	Living, dead	Frozen mammoth DNA (million-year-old)	No	No	Not sure if the cells contain DNA as genetic material	—	Possibility of non-DNA-type organisms	—
(27)				Fluorescence microscopy	Sample acquisition; staining; observation using a fluorescence microscope	—	20 kg	1 Cell	10 × 10 × 0.05 mm	1.3 × 10^3^ cells/g of soil	(27) 100%	100%	—	Living, dead	—	No	Abiotic organic globules, inorganic globules	Not sure if the cells contain DNA as genetic material	LDM	DNA existing but diffused, possibility of non-DNA-type organisms	—
(28)		ATP	1.5 amol (Gram-negative bacteria), 5.5 amol (Gram-positive bacteria)	Luciferin-luciferase luminescence assay	Sample acquisition; adding reaction medium; bioluminescence measurement	—	10 kg	3 Cells (Gram-negative bacteria), 1 cell (Gram-positive bacteria)	0.1 g	10^2^ cells/g of soil	(28) 100%	100%	—	Living, very fresh dead	—	No	No	Not sure if the cells contain ATP as energetic mediator	—	Possibility of organisms that do not use ATP	—
(29)	Metabolism	Catabolization		Labeled release experiment	Sample acquisition; addition of ^14^C-organic compounds; β-ray deletion	—	350 g	50 Colony forming units	0.5 g	10^4^ cells/g of soil	(29) 100%	Low	The determination of metabolic conditions and substrate is difficult	Living	—	No	Abiotic catalytic organic compounds, abiotic catalytic inorganics	High probability of having catabolization capability	Viking/RL	Just metabolism conditions are not suitable, dead organisms only	—
(8)				Scanning electron microscopy	Sample acquisition; sample preparation; metal coating or positive staining; observation using a scanning electron microscope	Metal coating or positive staining	50 kg	1 Cell	200 × 200 × 15 μm	1.1 × 10^5^ cells/g of soil	(8) 100%	100%	—	Living, dead	Extracellular proteins of mammoth (million-year-old)	—	Abiotic organic globules, inorganic globules	High probability of having a cellular structure	—	Irregular morphology (solo or aggregates) not identified as organisms, hidden organisms (*e.g*., in crack of mineral particle)	Imaging of organic and inorganic particles with high resolution (though they cannot be distinguished)
(9)				Atomic force microscopy	Sample acquisition; observation using an atomic force microscope	—	10 kg	1 Cell	200 × 200 × 15 μm	1.1 × 10^5^ cells/g of soil	(9) 100%	100%	—	Living, dead	—	—	Abiotic organic globules, inorganic globules	High probability of having a cellular structure	FAMARS	Irregular morphology (solo or aggregates) not identified as organisms, hidden organisms (*e.g*., in crack of mineral particle)	Imaging of organic and inorganic particles with high resolution (though they cannot be distinguished)
(10)	Cell constituent	Organic compound	0.3 pg	IR absorption	Sample acquisition; oxidation; IR measurement of carbon dioxide	—	35 kg	0.5 μg/mL	1 mL	10^6^ cells/g of soil	(10) 100%	100%	—	Living, dead	—	Abiotic organic compounds	No	High probability of having a cellular structure made of organic compounds	—	Quite severe environment where organic compounds are destroyed	Abiotic organic compounds
(11)				TV(Pyr)-GCMS	Sample acquisition; pyrolysis; GC/MS	—	50 kg	30 ng/g	100 mg	6 × 10^3^ cells/g of soil	(11) 100%	100%	—	Living, dead	—	Abiotic organic compounds	No	High probability of having a cellular structure made of organic compounds	Viking, SAM	Quite severe environment where organic compounds are destroyed	Abiotic organic compounds
(12)				Fluorescence microscopy	Sample acquisition; staining; observation using a fluorescence microscope	—	20 kg	1 Cell	10 × 10 × 0.05 mm	1.3 × 10^3^ cells/g of soil	(12) 100%	100%	—	Living, dead	—	Abiotic organic compounds	Abiotic organic globules, inorganic globules	High probability of having a cellular structure made of organic compounds	LDM	Organic compounds existing but diffused, quite severe environment where organic compounds are destroyed	Aggregates of abiotic organic compounds
(13)				LDI-MS	Sample acquisition; laser desorption; MS	—	100 kg	1 pmol/mm^2^	0.6 mm diameter × 0.05 mm	1.2 × 10^6^ cells/g of soil	(13) 100%	100%	—	Living, dead	—	Abiotic organic compounds	No	High probability of having a cellular structure made of organic compounds	SHERLOCK	Quite severe environment where organic compounds are destroyed	Abiotic organic compounds
(14)		Protein	0.2 pg	Colorimetric assay	Sample acquisition; cell destruction; partial purification; Bradford, Lowry, or BCA reaction; VIS measurement	Cell destruction (mechanical, freeze and thaw, *etc.*); partial purification (trichloroacetic acid precipitation, centrifugation, decantation, and dissolution)	0.3–5 kg	1 μg/mL	1 mL	5 × 10^6^ cells/g of soil	(14) 100%	100%	—	Living, dead	—	No	Abiotic amino acid condensates	High probability of having a cellular structure containing polymers of amino acids	—	Possibility of nonprotein-type organisms	—
(15)				Nitrogen quantification	Sample acquisition; cell destruction; partial purification; sulfuric acid hydrolysis; setting; ammonia quantification	Cell destruction (mechanical, freeze and thaw, *etc.*); partial purification (trichloroacetic acid precipitation, centrifugation, decantation and dissolution)	3 kg	0.1 g	Not applicable	5 × 10^11^ cells/g of soil	(15) 100%	100%	—	Living, dead	—	No	Abiotic amino acid condensates	High probability of having a cellular structure containing polymers of amino acids	—	Possibility of nonprotein-type organisms	—
(16)				UV absorption	Sample acquisition; cell destruction; partial purification; UV absorption at 280 nm	Cell destruction (mechanical, freeze and thaw, *etc.*); partial purification (trichloroacetic acid precipitation, centrifugation, decantation, and dissolution)	0.3–5 kg	50 μg/mL	1 mL	3 × 10^8^ cells/g of soil	(16) 100%	100%	—	Living, dead	—	No	Abiotic amino acid condensates	High probability of having a cellular structure containing polymers of amino acids	—	Possibility of nonprotein-type organisms	—
(17)				Fluorescence pigment staining	Sample acquisition; cell destruction; partial purification; adding CBQCA or NanoOrange; fluorescence measurement	Cell destruction (mechanical, freeze and thaw, *etc.*)	0.6–40 kg	10 ng/mL	1 mL	1.3 × 10^3^ cells/g of soil	(17) 100%	100%	—	Living, dead	—	No	Abiotic amino acid condensates	High probability of having a cellular structure containing polymers of amino acids	LDM	Protein existing but diffused, possibility of nonprotein-type organisms	—
(18)		Amino acid	0.2 pg	HPLC	Sample acquisition; cell destruction; water extraction; hydrolysis; LC; UV, fluorescence, or MS	Cell destruction (mechanical, freeze and thaw, *etc.*)	30 kg	10 fmol (100 cell)	0.1 g	10^3^ cells/g of soil	(18) 100%	100%	—	Living, dead	—	Abiotic amino acids	Compounds with similar retention time (almost “no” if LC/MS is applied)	High probability of having a cellular structure containing polymers of amino acids	—	Quite severe environment where amino acids are destroyed	Abiotic amino acids
(19)				Capillary electrophoresis	Sample acquisition; cell destruction; water extraction; hydrolysis; EP; UV, fluorescence, or MS	Cell destruction (mechanical, freeze and thaw, *etc.*)	35 kg	1 amol (1 cell)	0.1 g	10 cells/g of soil	(19) 100%	100%	—	Living, dead	—	Abiotic amino acids	Compounds with similar retention time	High probability of having a cellular structure containing polymers of amino acids	Urey	Quite severe environment where amino acids are destroyed	Abiotic amino acids

^a^ Spherical particles with similar radius, and rod-shaped structures with a continuous radius along the length whose size falls withing a specific radius, in a size range from submicrometer to submillimeter, are considered candidates of “cellular structures.”

^b^ By using an appropriate combination of pigments for detecting membrane permeability, it is possible to distinguish living from dead cells.

^c^ Dead cells can be detected while DNA is still present, and long RNA strands are dissociated after cell death. Thus, it is possible to distinguish them with FISH.

ATP = adenosine triphosphate; DNA = deoxyribonucleic acid; EP = electrophoresis; FISH = fluorescence *in situ* hybridization; HPLC = high-performance liquid chromatography; IR = infrared; LC = liquid chromatography; LC/MS = liquid chromatography mass spectrometry; LDI-MS = laser desorption ionization mass spectrometry; MS = mass spectrometry; RNA = ribonucleic acid; TV(Pyr)-GCMS = thermal volatilization or pyrolysis gas chromatography mass spectrometry; UV = ultraviolet; VIS = visible spectrometry.

Rather than repeating the explanation in Paper-I by describing each item through the entire table, this section reviews the summary table in comparison with the original version and concentrates discussion on the new items.

### 2.1. Table 1 columns (A) to (J): target characteristics and detection techniques

Table 1 columns from (A) to (J) focus on the target characterization, detection technique, and sensitivity. These are mainly unchanged as those presented in the summary table in Paper-I, except for minor editorial revisions. In Table 1, first two columns (A) and (B) list the major and subdivided detailed targeted characteristics for detecting microbial extraterrestrial life such as cell proliferation, cell morphology, and the cell constitutes (organic compounds, proteins, amino acids, nucleic acids that include deoxyribonucleic acid [DNA], ribonucleic acid [RNA], and adenosine triphosphate [ATP], and metabolism components such as catabolization molecules and enzymes). Table 1 column (C) lists the expected content of the targeted molecules in one cell.

The possible detection techniques are listed and explained in Table 1 columns (D)–(J). Table 1 columns (D) and (E) list each technique with a brief description, whereas Table 1 column (F) indicates any special pretreatment that would require human operation in ground-based experiments (either on Earth through a sample return, or on a crewed extraterrestrial base). Finally, Table 1 columns (G)–(J) report the instrument mass (for a ground-based instrument), the technique sensitivity for the target molecules, the required observed volume or mass for detection, and the sensitivity for cell density in this sample.

### 2.2. Table 1 column (K): generality for terrestrial microorganisms

In Paper-1, the “Generality for terrestrial microorganisms” was a single column listing a percentage success rate for detecting the target characteristic in a sample of terrestrial microbes. The single column made it difficult to ascertain the reason for a low value. For example, “metabolism” had a value of ca. 50% in Paper-1 due to the difficulty in detection despite being universally present in terrestrial microorganisms. We, therefore, expanded this property into five separate considerations surrounding that detection to allow a more nuanced discussion of the challenges involved beyond sample size.

#### 2.2.1. Table 1 column (Ka): probability of having the targeted characteristic

All targeted characteristics of extraterrestrial microbial life listed in Table 1 are general among terrestrial microorganisms. Although it is possible for individual terrestrial microorganisms to lose a characteristic, or the characteristic becomes dormant and, therefore, not presented during detection attempts, all terrestrial microorganisms as a species have all the targeted characteristics.

#### 2.2.2. Table 1 column (Kb): detection success rate and Table 1 column (Kc): essential difficulty

These second two subcategories consider the percentage success rate for detecting the target in a sample of terrestrial microbes (Kb) and briefly describe any challenges with the detection (Kc).

One of the chief challenges highlighted by these columns is the requirement of a suitable environment for the first target characteristic of the proliferation of cells. Determining adequate culture conditions for unknown microorganisms is difficult. Indeed, culturing terrestrial microorganisms has a reported success rate to date of just ≤1% (Amann *et al*., 1995; Pham and Kim, 2012).

Similarly, when targeting the chemical reactions constituting metabolism, it is also difficult to identify the adequate substrate and/or conditions that are used for detecting catalytic reactions and/or the metabolism of unknown organisms.

#### 2.2.3. Table 1 column (Kd): detectability of living or dead cells, and ability to distinguish between them

Table 1 column (Kd) indicates whether the detection technique can be used on living and/or dead cells. If both cell types can be detected whether the ability to distinguish the living cells from dead cells has been demonstrated is also recorded.

#### 2.2.4. Table 1 column (Ke): long-term detection

The final column in the comparison with terrestrial microorganism section tackles whether the target characteristics could still be detected in terrestrial microorganisms after an extended passage of time since the organism thrived. This is relevant when considering detection of life (extinct or suspended) after life-favorable conditions on the planet have passed.

The retention of both cell activity and the stability of the cell constituent macromolecules such as proteins and nucleic acid polymers depends strongly on the surrounding conditions, especially on water content and temperature.

Cell proliferation activity is lost within days after a liquid microbial culture reaches its death phase, which is the last growth phase after phases of lag (cells metabolically active but not dividing), log (exponential growth), and stationary (death equal to dividing cells). But microbial cells are often stored frozen in a deep freezer at −70°C or −80°C for years (Cody *et al*., 2008; Tedeschi and De Paoli, 2011). In the absence of liquid water, dried cells also retain their viability for years, and freeze-drying is used to store microbial strains in laboratories and in Culture Collection Centers. The dried cells of the radiation-resistant microorganism *Deinococcus radiodurans* survived for 3 years, whereas dried cell pellets are expected to survive for tens of years in the vacuum of space and in the dark based on extrapolating the survival fraction obtained over 3 years (Kawaguchi *et al.*, 2020).

In the natural (nonlaboratory) environment, a surviving cell of a hyper-halophile was recovered from a liquid inclusion embedded in crystal halite (Lowenstein *et al*., 2011). The recovery of live microorganisms from the inclusion points to the possible survival of microorganisms over long periods.

Similar examples exist for the cell constituent macromolecules. The presence of liquid water in cells allows the rapid progress of hydrolytic degradation of macromolecules, which are catalyzed by enzymes (*i.e*., hydrogenates) in temperatures above freezing. However, this suggests a survival time that is much longer below freezing and/or in desiccation conditions.

Proteins such as collagen have been discovered preserved in the bodies of mammoths from 40,000 years ago and can be inspected using transmission electron microscopy (TEM)-based spectroscopy and scanning electron microscopy (SEM) (Hattori *et al.*, 2021). The sequence of collagen has also been reported to have been recovered from the 68-million-year-old fossil bones of a *Tyrannosaurus rex* (San Antonio *et al*., 2011). DNA was also recovered from a 1-million-year-old frozen mammoth that was discovered in Siberia (van der Valk *et al*., 2021). However, it should be noted that all these analyses were performed using state-of-the-art equipment after a process of careful extraction of the materials from ground samples, which exceeds what robotic exploration analysis has achieved to date. Moreover, the samples in these analyses were exclusively from macroscopic specimens of large organisms. It remains uncertain whether it is possible to find examples of rock containing microfossils of micrometer-sized cells.

Organic molecules experience slow denaturation to form kerogen and graphite in sediment underground through dehydrogenation. Cellular microfossils have been found through inspection with an optical microscope in the sediment rock samples that have been cut, sliced, and polished. The oldest cell fossils were found in 3.5-billion-year-old rock, which was observed by optical microscopy and secondary ion mass spectrometry (SIMS) (Schopf *et al*., 2018).

Amino acids dating back billions of years have also been recovered from the Murchison carbonaceous chondrite meteorites through liquid extraction and analyzed with gas chromatography-mass spectrometry (GC-MS) after derivatization (Koga and Naraoka, 2017; Heck *et al*., 2020).

This evidence of ancient terrestrial microbial cells indicates that desiccated and/or frozen conditions allow living organisms to be retained for tens of years, and macromolecules to be retained for billions of years. However, in the presence of liquid water above freezing temperatures cause both living cells and macromolecules to be lost quickly.

### 2.3. Table 1 column (L): source of false positives (with the exception of Earth contamination)

The occurrence of a false positive can occur when an abiotic process resembles or replicates the target characteristic of a microorganism when observed with certain detection techniques. Possible sources of false positives were previously listed in a single column in the original table of Paper-I. To understand this issue more thoroughly, this attribute is now split into Table 1 two columns: (La) concerns the possibility of the target characteristic being generated by a nonliving process, whereas (Lb) lists processes that could resemble the target characteristic during particular detection methods.

#### 2.3.1. Table 1 column (La): possibility of the targeted characteristics being generated by nonlife

##### 2.3.1.1. Cell proliferation

Proliferation is the reproduction of individual sources of life, and the word “proliferation” contains the term “life” itself. In this article, it is accordingly considered impossible for a nonliving source to proliferate. The Table 1 column (La) for proliferation, therefore, records “no.”

##### 2.3.1.2. Cell morphology

The form and structure of a cell provides important information in the search for examples of extraterrestrial life. In terrestrial life, coccoid cells of the same species usually have a similar radius. Similarly, rod-shaped cells typically have a continuous one-cell radius along the length whose size falls within a specific radius range within each species. Nevertheless, morphology alone cannot be the sole factor in judging whether the observed object constitutes life since abiotic structures can also form similar morphologies. It is, therefore, desirable to combine morphology with other observed characteristics, such as the cell constituents. Sources of morphologies that may resemble biological structures are described in Table 1 column (Lb), and Table 1 column (La) is “—.”

##### 2.3.1.3. Organic compounds

It is well established that organic compounds can be generated abiotically. More than 200 molecular species, excluding isotopologues, have been identified in interstellar clouds and circumstellar shells (Guélin and Cernicharo, 2022), and 1000 organic molecules have been detected in carbonaceous chondrites (Schmitt-Kopplin *et al*., 2010; Zherebker *et al*., 2021). These organic molecules were brought to the planets in our solar system, including the Earth, during and after their formation.

##### 2.3.1.4. Amino acids

There are also extensive examples of amino acids generated in environments outside biological systems. More than 90 species of amino acids have been detected in carbonaceous chondrites (Glavin *et al*., 2020). The simplest amino acid glycine has also been found in samples from a comet coma returned by the NASA *Stardust* mission (Elsila *et al*., 2010). As with other organic compounds, these discoveries indicate that meteorites would have delivered amino acids to the Earth and other planets in the Solar System early in their histories.

##### 2.3.1.5. Proteins

The structure and catalytic activity of proteins is known to have arisen during the Darwinian evolution of living organisms (Alberts, 2015). Proteins, therefore, are not produced abiotically.

##### 2.3.1.6. Nucleic acids

Abiotic nucleic acids have never been detected in natural environments. However, laboratory experiments suggest that abiotic synthesis of nucleic acids could exist (Ayukawa *et al*., 2019). The possibility of a nonbiological origin should, therefore, be considered.

##### 2.3.1.7. Deoxyribonucleic acid

Abiotic DNA has also never been found in natural environments. Unlike RNAs, there are also no experiments suggesting that DNA could be synthesized in nonbiological processes.

##### 2.3.1.8. Adenosine triphosphate

As with DNA, naturally formed abiotic ATP has never been found and there are no known experimental pathways.

##### 2.3.1.9. Catabolization

Metabolism is defined by the catalysis carried out by life. That is, metabolism cannot be carried out by a nonliving system. As with cell proliferation, Table 1 column (La) in this case is, therefore, “no.”

##### 2.3.1.10. Enzymes

As with proteins, the structure and catalytic activity of proteinous catalysts first appeared during Darwinian evolution of living organisms. Enzymes can, therefore, not be produced abiotically.

#### 2.3.2. Table 1 column (Lb): possibility of detecting nontargeted characteristics

In addition to the possibility of abiotic creation of the targeted characteristics, there is the related issue of detecting a different process with similar features. This second type of false positive is considered in Table 1 column (Lb).

In liquid culture needed for detection of cell proliferation, crystallization, or precipitation of inorganic molecules can occur and represent false-positive sources of replication.

For cell morphology, various microscopic structures such as abiotic organic globules and inorganic globules could potentially be mistakenly identified as morphological observations of a cell.

Cell constituents can also fall victim to false detections, depending on the method employed. In the techniques used for detecting proteins, abiotic amino acid condensates can yield false positive results. Compounds with similar retention time are false positive sources in detecting amino acids using high-performance liquid chromatography (HPLC), capillary electrophoresis, and laser desorption ionization mass spectrometry (LDI-MS). However, almost all false positives can be avoided for HPLC if liquid chromatography mass spectrometry (LC/MS) is applied. When using ultraviolet (UV) absorption measurements to detect nucleic acids and DNA, other compounds with absorption at 260 nm can be false positive sources, but these can be identified to some extent by using a precise spectrum analysis.

In metabolism detection, abiotic catalytic organic compounds such as ribozymes and proteinoids, and abiotic catalytic inorganic such as clay, Fe-S, Fe ions, and Zn ions are false positive sources in radiation measurements and in enzyme activity assays.

The application of fluorescence microscopy in detecting various targeted characteristics, as listed in Table 1, can produce false positives for abiotic organic globules and inorganic globules, depending on the fluorescent pigment used.

### 2.4. Table 1 column (M): universality of targeted characteristics

What is the plausibility that extraterrestrial life will exhibit the target characteristics? This column was not previously considered in Paper-I and considers how universal the target characteristics are likely to be in independent occurrences of life based on abundance of basic materials and variations on Earth.

#### 2.4.1. Organic compounds

As was previously discussed for Table 1 column (L), it is well established that organic compounds can be generated abiotically. With the ability to bond to four neighboring atoms, carbon can form linear molecules with side chains that can carry a wide variety of functional groups. This characteristic allows carbon to form the backbone in biological molecules, and to retain genetic information and catalytic function in terrestrial life when combined with several other atom types.

Silicon has been proposed as an alternative atom for the functionality of carbon that could be utilized by other life-forms in the universe. However, it is worth noting that the majority of silicon-containing compounds found in natural environments on Earth and in space are silicates, owing to the extremely stable bond between silicon and oxygen (Greenwood and Earnshaw, 1997). While artificial silicon compounds are synthesized and used in industry, the molecules are made up of carbon in addition to silicon (Moretto et al., 2000).

The characteristics of carbon and the ubiquitous presence of organic molecules in space suggests that extraterrestrial microorganisms independent from life on Earth would be highly likely to consist of carbon-based molecules, that is, organic compounds.

#### 2.4.2. Amino acids

Amino acids are also compounds that can be generated in nonbiological environments. Amino acids with various side chains are easily polymerized through heat-based dehydration to form proteinoids (Fox and Harada, 1958). In biological systems, such polymers of amino acids that can be folded into specific structures with highly specific catalytic activities were created through natural selection, after the emergence of the genetic system.

The formation of linear polymer molecules with a variety of side chains can be potentially formed with other types of molecular bonds, such as ether bonds, carboxylic-acid-ether bonds, or dieter bonds through inorganic acids. However, few species of monomeric molecules that could form such heteropolymers have been found in space or in carbonaceous chondrites (Schmitt-Kopplin *et al.*, 2010; Zherebker *et al.*, 2021; Guélin and Cernicharo, 2022). Therefore, if life were to be found elsewhere in the Solar System, there is a high probability that such life-forms will use amino acids to form catalytic polymer molecules such as proteins.

#### 2.4.3. Proteins

The only polymers that have been shown to date to exhibit catalytic activity are proteins and RNA. Protein is a polymer of amino acids. The presence of many species of amino acids with various side chains allows for the formation of catalytic polymers. It has been proposed that, in the early stages of life on Earth, inorganic atoms such as iron and sulfur exerted catalytic functions. However, even in such “metabolism-first” hypotheses, inorganic catalysts were replaced by proteins that contained the iron and sulfur as catalytic centers in the later evolutional stage (Martin and Russell, 2003). This is because the catalytic proteins (*i.e*., enzymes) have very high catalytic and substrate specificities, as well as a catalytic activity that is often several orders of magnitude higher than that of the inorganic molecules.

It is also important to note that many amino acid species have been found in carbonaceous chondrites and that these would have supplied these amino acid species to the surface of celestial bodies throughout the Solar System. Although nuclear bases and ribose have also been detected in carbonaceous chondrites, no ribonucleosides or monomers of RNA have been found in meteorites. The universal presence and, therefore, usage of ribozymes are not guaranteed in extraterrestrial environments.

If life-forms are discovered elsewhere in the Solar System, it seems likely that there is a high probability of carrying catalytic polymer molecules consisting of amino acids such as proteins.

#### 2.4.4. Nucleic acids, DNA, and ATP

Unlike the case for proteins and amino acids, it is not certain whether life forming outside the Earth would contain nucleic acids or DNA as genetic material, or ATP as an energetic mediator. Nucleic acids have never been detected in meteorites, even though nucleobases have been found in carbonaceous chondrites (Callahan *et al*., 2011). It has been demonstrated that polymers that have a main chain connected with peptide bonds and a side chain of nucleobases can hybridize (Lee *et al*., 2007). Alternative types of generic polymers, if these were to be found on other planets or satellites, may, therefore, act as the genetic material for extraterrestrial life.

It is also highly significant that the canonical nucleobases, that is, adenine, cytosine, guanine, uridine, and thymine are not the only nucleobases that can exist. Many other base pairs have been replicated in the laboratory (Mitsui *et al*., 2003; Benner, 2004; Hirao *et al*., 2006), suggesting the existence of genetic systems that can use non-Watson-Crick-type base pairs.

In terrestrial biological cells, nucleoside triphosphate (NTP) species other than ATP are used in metabolic synthetic pathways. ATP, CTP, GTP, and UTP are used, respectively, in energetic systems, lipid synthesis, translation, and carbohydrate metabolism (Voet and Voet, 2010). All NTP species can retain Gibbs energy and participate in metabolic reactions. Thus, any of the nucleoside-triphosphate species, including ATP, CTP, GTP, or UTP, could be the energy-carrying molecules for life-forms in space.

### 2.5. Table 1 column (N): instruments for space missions

This column lists instruments on current space missions that have (or are being) used to detect the target characteristic. The list is the same as that presented in Paper-I.

### 2.6. Table 1 column (O): possible meaning of negative detection

One of the challenges when developing a scientific program is how to interpret a negative result. This is especially true for first detection projects such as extraterrestrial life searches, where there is large uncertainty in the form of the detectable phenomena. If an instrument returns a null result, does this categorically confirm that no life exists (and there is no purpose to future exploration) or could there be additional reasons why a negative result would occur even in the presence of life? The answer is essential for maintaining project value.

The new addition of this attribute to Table 1 considers the sources of negative detections, other than the obvious case of the absence of detectable life, to maximize understanding of the expected data.

#### 2.6.1. Cell proliferation

The success probability of a culture is quite low due to the difficulty in determining proliferation conditions even for the known cases of terrestrial microorganisms, as recorded in Table 1 columns (Ka) and (Kb). A negative result is, therefore, highly likely and may simply mean that the culture conditions were not suitable. Alternatively, microorganisms may exist but be deceased.

#### 2.6.2. Cell morphology

A negative detection in morphology observations in the presence of life can originate from a number of possibilities. For example, irregular morphology (solo cells or aggregates) may not be identified as organisms, or the organisms may be hidden, such as the crevices within mineral particles.

In the case of optical or fluorescence microscopy, it is also possible that a negative detection may arise from existing organisms not being stained by the pigment used for the observation. A negative detection in Raman spectroscopy or UV fluorescence spectroscopy could also mean that the organisms did not possess the Raman or fluorescence signal used in the observation.

#### 2.6.3. Organic compounds

Terrestrial organisms are mainly composed of water and organic compounds, which makes organic compounds a promising target for detecting life in extraterrestrial environments. However, a wide variety of organic compounds have been detected in carbonaceous chondrites (Kvenvolden *et al*., 1970), comets (Kissel and Krueger, 1987), and asteroids (Nakamura *et al*., 2022), which are considered to have formed abiotically.

Before the origin of life on Earth, organic compounds must have been carried to the surface of the Earth by meteorites, micrometeorites, comets, and/or interstellar dust. The detection and analysis of organic compounds on the surface of extraterrestrial celestial bodies is, therefore, also important, even if these compounds are generated abiotic. Analysis of organics will clarify our understanding of their origin and the way they were transferred to the surface of the celestial body. Moreover, their detection will elucidate the amount and type of organic compounds that were transferred and whether they were used in the emergence of life.

In the case of absence of detectable amounts of organic compounds, potential explanations should be considered. In the Viking mission, the TV-GC/MS experiment did not detect organic molecules on the martian surface. This negative result suggested that the martian regolith might hold a potent oxidant that converts all organic molecules rapidly to carbon dioxide relative to their arrival rate (Benner *et al.*, 2000).

#### 2.6.4. Amino acids

Amino acids are components of proteins. However, their detection in carbonaceous chondrites demonstrates that these can be formed abiotically (Kvenvolden *et al*., 1970; Martins *et al*., 2008; Furukawa *et al*., 2019). The type and chirality of amino acids could inform on their origin; terrestrial organisms generally use 20 amino acids with left-handedness, whereas carbonaceous chondrites have contained a much greater variety of racemic amino acids (Glavin *et al.*, 2020). The analysis of chirality and species can provide important information regarding the potential biological origin of any detected amino acids.

The detection of nonbiological origin biomolecular monomers would not allow the declaration of the presence of life, but possible abiotic formation pathways or delivery of such molecules should be discussed. Identification of the various types of racemic amino acids can assist in determining which space bodies, such as carbonaceous chondrites, contributed to the delivery of organic compounds to the surface of planets.

#### 2.6.5. Proteins, nucleic acids, and ATP

The presence of certain bio-organic molecules, such as proteins, nucleic acids, and ATP, would constitute direct evidence of terrestrial-type organisms given their abiotic synthesis is difficult. A failure to detect these would nevertheless be a starting point for the search for molecules composed of nonterrestrial-type organisms (Cafferty *et al*., 2018).

As previously discussed, proteins, nucleic acids, and ATP are not the only molecules that support catalysis, genetic information, and energy; they would be the starting point of a search for alternative molecules that can carry out these functions in situations where other methods (such as morphological analyses) detected a candidate organism that suggested the presence of life.

#### 2.6.6. Metabolism

The identification of metabolic conditions and substrates is difficult, as previously noted in Table 1 column (Kc). A negative result is, therefore, highly likely in the radiation measurement of catabolization and may simply imply that the metabolism conditions were not suitable. Alternatively, microorganisms may exist but are dead.

A negative detection in the case of catalytic activity assays for enzymes possibly means that the organisms were present but did not use the enzyme in question, that the reaction conditions were not suitable, or that the enzyme existed but was diffused. In fluorescence microscopy, a negative detection where other methods (such as morphological analyses) detected a candidate organism suggestive of the presence of life could likewise mean that the organism existed but did not use that enzyme or that the reaction conditions were unsuitable.

### 2.7. Table 1 column (P): science objectives other than the search for life

In addition to information regarding the existence of life that might be discovered in a negative detection, alternative scientific goals might be achieved from the results that do not directly pertain to extraterrestrial organisms. This column in Table 1 is a new addition to help maximize the data use from these experiments.

In Table 1, four types of microscopy (optical, fluorescence, Raman, and UV) are listed as techniques to identify cell morphology. Even if no life is present, organic components carried from space, such as carbonaceous chondrites, could be detected as particles or lumps of fragments. Moreover, imaging inorganic particles is also possible with these microscopies. The use of TEM, SEM, and atomic force microscopy (AFM) allows the imaging of organic and inorganic particles at high resolution. However, it is not possible to classify these using high-resolution microscopy techniques to determine whether the particles are inorganic, organic, proteins, nucleic acids, or otherwise.

The techniques listed in Table 1 for detecting cell constituents may discover abiotic amino acids and nucleic acids, which are important scientific discoveries even in the absence of life. Similarly, in methods for detection of enzymes, both catalytic organic compounds and catalytic inorganic compounds are scientifically valuable finds regardless of whether life is present.

## 3. Discussion

Section 2 revised the summary table originally presented in Paper-I for detecting different target characteristics of microorganisms. This section now discusses the search for extraterrestrial life based on the extended table.

### 3.1. Earth life, Earth-kin life, and Earth-independent life

It is helpful when discussing the detectability of extraterrestrial life to divide potential discoveries into three possible categories.

(1) Earth life: The discovery of Earth life is that of microorganisms that are the same species as those on Earth. Such a detection would usually be expected to be due to Earth contamination. Variations that may have evolved from the common ancestor in extraterrestrial environments are considered here in the separate category of “Earth-kin life.”
(2) Earth-kin life: This is extraterrestrial life that shares a common ancestor with life on Earth. It is not due to contamination from current life on Earth (see previous category) but also not an independent emergence of life from abiotic processes. Earth-kin life includes cases related to the panspermia hypothesis in which life migrated from Earth to Mars in ancient times, migrated in the reverse direction, or life from the same species or a shared common ancestor that originated in a third place within the universe arrived on both Earth and Mars (Arrhenius, 1908).
(3) Earth-independent life: This final category is an entirely separate emergence of life whereby extraterrestrial life began from nonliving systems entirely independently from life on Earth.

The targeted characteristics presented in Table 1 columns (A) and (B) are fundamental properties of Earth life. They are common to all life on Earth and are not easily replaced by other molecules, as noted in Table 1 columns K(a–e). Their universality suggests that the common ancestor to Earth life must also have displayed these characteristics and are, therefore, likely shared by Earth-kin life, which evolved from that same ancestor. All the techniques that are widely effective in the detection of Earth life thus have a high potential to detect Earth-kin life. Conversely, the applicability of such techniques to the detection of Earth-independent life is not guaranteed, as considered in Table 1 column (M) on the universality of targeted characteristics among Earth-independent microorganisms. This means that when discussing the design and data from extraterrestrial life searches, it is important to distinguish between discoveries of Earth-kin life and Earth-independent life. Similarly, Earth life also must be distinguished from Earth-kin life to distinguish from space vehicle-born contamination. This is discussed below in Section 3.4.

### 3.2. Re-evaluation of the conclusions of the previous research

Based on the previous analysis of the original version of Table 1 in Paper-I, the following conclusions about the search for extraterrestrial life were noted:

(1) There is no realistic single detection method that can conclusively determine the discovery of life independent of Earth.
(2) There is no single best technique for detecting characteristics of extraterrestrial life, which is superior to all other techniques in all respects.
(3) There is no single technique that can distinguish between Earth life and life independent of Earth.

Reconsideration of (1) recognizes that this is an ambiguous statement, as it could refer to either (a) the discovery of life or (b) the demonstration that life is Earth-independent. The latter option (b) is equivalent to point (3). Option (a) is, therefore, the more relevant interpretation. To consider the power of a detection method to discover life, it is important to consider the possible false positives to avoid declaring examples of nonlife as life. In the extended version of Table 1 presented in Section 2, false positives are resolved into Table 1 two columns, L(a) and L(b), to provide further details. This shows us that false positives are possible in all methods, except for solid cultures, DNA sequencing, and ATP measurements. It is worth noting that techniques requiring cultures are inherently difficult, due to the extreme difficulty of determining the culture conditions [see Table 1 columns K(b) and K(c)]. With regard to DNA sequencing and ATP detection, there is no guarantee that Earth-independent life carries these molecules (Table 1 column M). Accordingly, it remains true that there is no realistic single detection method that can conclusively determine the discovery of life, regardless of whether that life is Earth life or not. Point (2) also clearly still holds when considering the updated Table 1. As highlighted earlier, each technique has inherent advantages and disadvantages regarding false positive results [Table 1 columns L(a) and L(b)], generality in terrestrial microorganisms [Table 1 columns K(a) to K(e)] or universality beyond terrestrial life (Table 1 column M). The situation is similar to the comparison of the contents listed in Table 1 column (F) for special pretreatment requiring human operation in ground-based experiments, Table 1 column (G) for instrument mass (ground-based), Table 1 column (H) for sensitivity of the targeted characteristics, Table 1 column (I) for observation volume or mass, and Table 1 column (J) for sensitivity (cell density). There is, therefore, still no single measurement that is superior to all others in all respects.

When considering distinguishing between Earth life and Earth-independent life in point (3), the expanded version of Table 1 column (K) is helpful. One hundred percent of Earth life species have the targeted characteristics listed in Table 1, and, moreover, all the techniques listed can detect Earth life (although the success rate of culture methods and metabolic detection are low due to the difficulty in determining the conditions). Without an example of an attribute shown by extraterrestrial life and non-Earth life, no single method presented in Table 1 can distinguish Earth life from life independent from Earth.

These three conclusions, therefore, remain unchanged in this study.

### 3.3. Combination of techniques

As no single method can conclusively determine the presence of life, the next consideration is what combinations of methods might yield the most informative result. The number of possible combinations of techniques is clearly vast with a varied range of advantages. However, combinations with imaging are interesting since the majority of techniques in Table 1 aim to detect molecules without microscopic spatial resolution of the analyzed sample. Combining the methods that detect molecules with observations that have microscopic spatial resolution can provide an indication as to whether the detected molecules are widely and thinly distributed in the sample or localized to the size of a cell (or cell aggregate). The estimation of morphology combined with the analysis of molecules is proposed as a highly valuable technique combination.

#### 3.3.1. Comparison of imaging methods

The imaging techniques presented in Table 1 are optical microscopy, fluorescence microscopy, Raman microscopy, UV fluorescence microscopy, TEM, SEM, and AFM. Among these methods, fluorescence microscopy has a number of unique features; it is capable of not only imaging various molecules, but also detecting these different molecules using different fluorescent pigments. Imaging combined with molecular detection using fluorescence microscopy can, therefore, be performed simultaneously within a single field of view. Fluorescence microscopy also provides high sensitivity for molecular detection and high spatial resolution. Although the spatial resolution is lower than that of TEM, SEM, and AFM, fluorescence microscopy provides a much wider single-shot field of view compared with the ultrahigh-resolution microscopy techniques.

#### 3.3.2. Combination of mass spectrometry and fluorescence microscopy

A combination of particular interest is that of mass spectrometry (MS) with fluorescence microscopy. These two techniques provide complementary capabilities (previously described in Paper-I) as fluorescence microscopy provides morphological and rough information regarding the cell constituents, whereas MS offers detailed information on the molecules. Recently, identifying molecules as biosignatures using assembly theory and MS has been proposed (Marshall *et al*., 2021). These types of new analytical methods will increase the value of combining MS with other techniques such as microscopy.

### 3.4. The multiple steps of extraterrestrial life exploration missions

Given the difficulty of determining the existence of extraterrestrial life when using a single method, it can be beneficial to divide an extraterrestrial life exploration mission into multiple steps.

#### 3.4.1. Survey-and-detection step

This first task in an exploration mission is to survey a region and identify possible candidates for extraterrestrial life. This requires a detection method with a high sensitivity but also the capacity to check a large number of samples. Imaging using a wide field of view is effective in this setting, but ultrahigh-resolution imaging such as that of SEM, TEM, or AFM, would not be required in this step. Moreover, obtaining approximate information on morphology at this stage is worthwhile, as well as is determining whether the observed targets look like micron-size points, or are diffused over a wider area, since the former are more likely to be microbial cells. It is also essential to detect microorganism candidates without overlooking possible targets, even if that requires toleration of false positives (Paper-I). Fluorescence imaging is a strong candidate for a detection technique in this step, owing to the wide field of view.

#### 3.4.2. Analysis-and-conclusion step

**FIG. 1.**
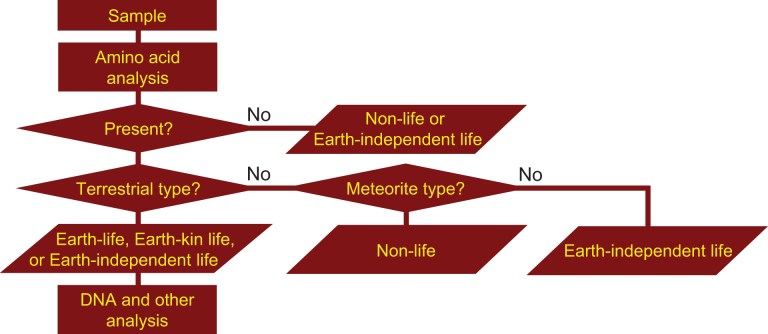
Flowchart for judging the origin of organisms through amino acid analyses (modified from Enya et al., 2022b). If amino acids could not be detected, the organism is judged to be either nonlife or Earth-independent life. If amino acids are found but differ from terrestrial proteinogenic types, they can be compared with those found in meteorites to ascertain they are nonlife (if matching) or Earth-independent life if differing. DNA and other analyses can be further performed to differentiate between Earth, Earth-kin, and Earth-independent life. DNA, deoxyribonucleic acid.

**FIG. 2.**
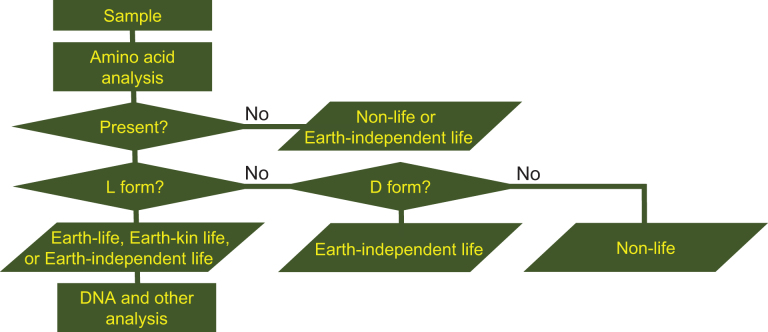
Flowchart for judging the origin of organisms through analysis of the enantiomeric excess of amino acids (modified from Enya et al., 2022b). If amino acids could not be detected, the organism is judged to be either nonlife or Earth-independent life. If amino acids are found and they are D-form, the organism is Earth-independent life. The organism is nonlife if the amino acids are a racemic mixture (not L- nor D-form alone). DNA and other analyses can determine further to judge if the organism is Earth, Earth-kin, or Earth-independent life.

**FIG. 3.**
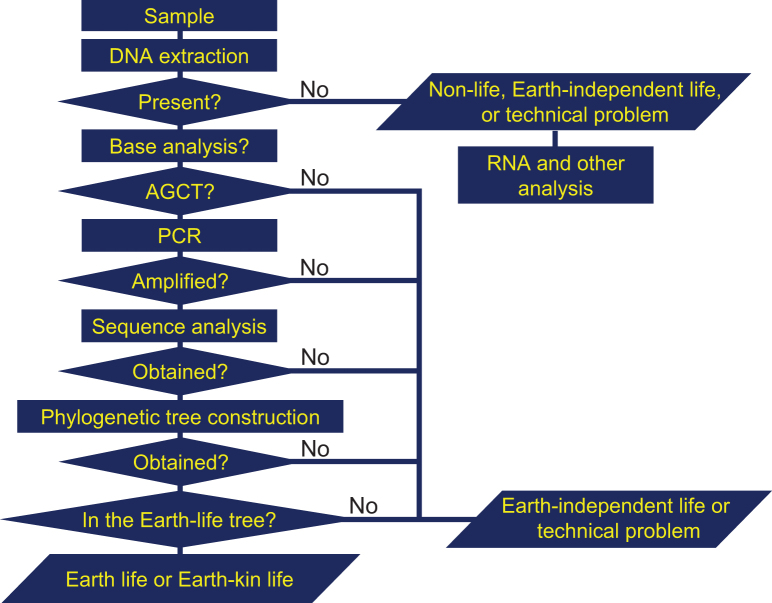
Flowchart for judging the origin of organisms through DNA analyses (modified from Enya et al., 2022b). The absence of DNA suggests either nonlife, Earth-independent life, or a technical issue. Subsequent DNA-base analysis can be used to test the presence of ACGT. PCR analysis can test if the DNA strand can be amplified and if amplification is successful, the sequence can be analyzed and a phylogenetic tree constructed. The failure in these procedures suggests that the organism is Earth-independent life or technical problem. The position of the sequence obtained in the tree would indicate how the organism is related to terrestrial organisms, and the time in the history when the branch away from terrestrial life occurred, so that we can tell if it is Earth or Earth-kin life. ACGT, adenine, cytosine, guanine, and thymine; PCR, polymerase chain reaction.

If microorganisms are identified within the observation region, the next process is a detailed characterization of the constituents and molecules within the candidate organisms, such as the existence of proteins, nucleic acids, DNA, amino acids, ATP, and enzymes. Subsequent processes in this step include analysis aimed at distinguishing the microorganism candidates from terrestrial organisms that might still be present as contamination on the instrument. The three flowcharts, Figs. 1–3 demonstrate this process for the case of amino acids or DNA analyses. From Figs. 1–3, it is obvious that distinguishing between the three types of life (Earth, Earth-kin, and Earth-independent) is not trivial nor is determining life versus nonlife. Detailed analysis is required to distinguish between the different types of organic particles. The requirement for such an in-depth study also rationalizes the need to separate extraterrestrial life exploration missions into the survey-and-detection and analysis-and-conclusion steps, and to first conduct the survey-and-detection step without overlooking the possibility of life (organic particles).

In this analysis-and-conclusion step, imaging observations are again worthwhile. But compared with the survey-and-detection step, higher spatial resolution is now required to perform more detailed morphological observations.

After the identification of candidate characteristics and their environmental conditions, it may be worthwhile to attempt to culture the candidates to obtain conclusive evidence for life.

The functional choice for the analysis will also depend on the analysis *situation*, such as *in situ* robotic operations during Solar System exploration, analysis of a returned sample on Earth, and crewed work that probably utilizes an extraterrestrial base. The analysis “situation” is a separate concept from the analysis “step” described earlier. For instance, it is reasonable to initially conduct the survey-and-detection step as an *in situ* survey with a robotic explorer and to follow this in the analysis-and-conclusion step that involves returned samples in a ground-based laboratory or at an extraterrestrial base. Alternatively, it would also be possible to try an *in situ* analysis for the analysis-and-conclusion step or to perform the survey-and-detection step from an extraterrestrial base, depending on future circumstances.

The results obtained in the survey-and-detection step will provide essential information that will influence the design of exploration missions and the instruments used in the second step. These two steps are examples, and many variations are of course possible in designing approaches to conclusively detect extraterrestrial life, which may include three or more steps.

As discussed in this section, imaging instruments can be a useful or even essential tool in any designed step to identify extraterrestrial microorganisms, whereas the optimal instrument design could vary based on the step and situation.

### 3.5. Scientific objectives beyond life detection

When developing a space exploration mission or instrument for extraterrestrial life detection, it is important to consider positive outcomes in the eventuality that no extraterrestrial life is found. A plan with zero outcomes upon finding no life will be much less likely to result in the proposed mission or instrument being adopted.

For this reason, the extension of Table 1 in this study includes the two new columns, “(O) Possible meanings of negative detection” and “(P) Scientific objectives other than life detection.” These additions demonstrate that, although it is undesirable to detect characteristics generated abiotically (abiotic organic compounds, abiotic amino acids, *etc.*) if the intention was solely to identify life, the capacity to detect abiotic characteristics provides the ability to elucidate alternative aspects, that is, scientific studies with objectives other than finding life itself.

For example, the potential discovery of abiotic amino acids could be key for mapping the delivery of organic compounds to terrestrial planets or understanding abiotic chemical processes that do not occur in an Earth-like environment. Identifying aggregates of abiotic organic compounds could point to nonterrestrial pathways that might be utilized by Earth-independent life.

Such goals are especially important as it is quite possible that an extraterrestrial life search mission will not result in the identification of life. In terms of the programmatic as well as the scientific sense, it is therefore important in realizing a space exploration mission or instrument for extraterrestrial life searches to design a plan such that scientific results can be obtained even if no life is found. In this situation, resolving the morphological images of particles of regolith, grinded powder of rock, or aerosols is of interest, because these can provide a base for judging what can be expected and planned for in later projects, both scientifically as well as for technical detection considerations. Additional information regarding the characteristics of the particles will be useful and can be achieved with microscopes, especially with fluorescence microscopy. The information in the microscopic images will be expanded using the molecular information obtained from a mass spectrum.

The targeted characteristics and techniques described in this article are valid for a wide range of celestial bodies even though optimization of instrument design and sample acquisition systems will be specific to the destination. These include Mars, Venus, and other planets; icy moons such as Europa, Ganymede, Enceladus, and Titan; non-icy moons such as Phobos, Deimos, and our Moon; small bodies, and so on. Furthermore, these targeted characteristics and techniques are important subjects in the research of microorganisms in Earth's environment, and it is possible that our planet's microorganisms can be used to demonstrate and update the methods that can be used for extraterrestrial life exploration.

## Notes

Associate Editor: Jorge Vago

## Abbreviations Used

ACGT: adenine, cytosine, guanine, and thymine
AFM: atomic force microscopy
ATP: adenosine triphosphate
DNA: deoxyribonucleic acid
EP: electrophoresis
FISH: fluorescence *in situ* hybridization
HPLC: high-performance liquid chromatography
IR: infrared
LC: liquid chromatography
LC/MS: liquid chromatography mass spectrometry
LDI-MS: laser desorption ionization mass spectrometry
MS: mass spectrometry
NTP: nucleoside triphosphate
PCR: polymerase chain reaction
RNA: ribonucleic acid
SEM: scanning electron microscopy
TEM: transmission electron microscopy
TV-GC/MS: thermal volatilization gas chromatography/mass spectrometry
TV(Pyr)-GCMS: Thermal volatilization or pyrolysis gas chromatography mass spectrometry
UV: ultraviolet
VIS: visible spectrometry

## References

[B1] Alberts B. Molecular Biology of the Cell (6th ed.). W.W. Norton & Company: New York; 2015.

[B2] Amann RI, Ludwig W, Schleifer K-H. Phylogenetic identification and *in situ* detection of individual microbial cells without cultivation. Microbiol Rev 1995;59(1):143–169. 7535888 10.1128/mr.59.1.143-169.1995 PMC239358

[B3] Arrhenius S. Worlds in the Making: The Evolution of the Universe. Harper & Brothers: London and New York; 1908.

[B4] Ayukawa S, Enomoto T, Kiga D. RNA World. In: Astrobiology: From the Origins of Life to the Search for Extraterrestrial Intelligence. (Yamagishi A, Kakegawa T, Usui T. eds.) Springer Singapore: Singapore, 2019; pp. 77–90.

[B5] Benner SA. Understanding nucleic acids using synthetic chemistry. Acc Chem Res 2004;37(10):784–797; doi: 10.1021/ar040004z 15491125

[B6] Benner SA, Devine KG, Matveeva LN, *et al.* The missing organic molecules on Mars. Proc Natl Acad Sci U S A 2000;97(6):2425–2430. 10706606 10.1073/pnas.040539497 PMC15945

[B7] Biemann K, Oro J, Toulmin P, III, *et al.* The search for organic substances and inorganic volatile compounds in the surface of Mars. J Geophys Res 1977;82(28):4641–4658; doi: 10.1029/JS082i028p04641

[B8] Cafferty BJ, Fialho DM, Hud NV. Searching for possible ancestors of RNA: The self-assembly hypothesis for the origin of proto-RNA. In: Prebiotic Chemistry and Chemical Evolution of Nucleic Acids. (Menor-Salván C. ed.) Springer International Publishing: Cham, 2018; pp. 143–174.

[B9] Callahan MP, Smith KE, Cleaves HJ, *et al.* Carbonaceous meteorites contain a wide range of extraterrestrial nucleobases. Proc Natl Acad Sci U S A 2011;108(34):13995–13998; doi: 10.1073/pnas.1106493108 21836052 PMC3161613

[B10] Cody WL, Wilson JW, Hendrixson DR, *et al.* Skim milk enhances the preservation of thawed −80°C bacterial stocks. J Microbiol Methods 2008;75(1):135–138; doi: 10.1016/j.mimet.2008.05.006 18573555 PMC2551311

[B11] Drake FD, Sobel D. Is anyone out there. Mercury 1992;21:120.

[B12] Elsila JE, Glavin DP, Dworkin JP. Cometary glycine detected in samples returned by Stardust. Meteorit Planet Sci 2010;44(9):1323–1330; doi: 10.1111/j.1945-5100.2009.tb01224.x

[B13] Enya K, Yamagishi A, Kobayashi K, *et al.* Comparative study of methods for detecting extraterrestrial life in exploration mission of Mars and the Solar System. Life Sci Space Res 2022a;34:53–67; doi: 10.1016/j.lssr.2022.07.001 35940690

[B14] Enya K, Yoshimura Y, Kobayashi K, *et al.* Extraterrestrial life signature detection microscopy: Search and analysis of cells and organics on Mars and other Solar System bodies. Space Sci Rev 2022b;218(6):49; doi: 10.1007/s11214-022-00920-4

[B15] Fox SW, Harada K. Thermal copolymerization of amino acids to a product resembling protein. Science 1958;128(3333):1214–1214; doi: 10.1126/science.128.3333.1214 13592311

[B16] Fujii Y, Angerhausen D, Deitrick R, *et al.* Exoplanet biosignatures: Observational prospects. Astrobiology 2018;18(6):739–778; doi: 10.1089/ast.2017.1733 29938537 PMC6016572

[B17] Furukawa Y, Chikaraishi Y, Ohkouchi N, *et al.* Extraterrestrial ribose and other sugars in primitive meteorites. Proc Natl Acad Sci U S A 2019;116(49):24440–24445. 31740594 10.1073/pnas.1907169116 PMC6900709

[B18] Glavin DP, McLain HL, Dworkin JP, *et al.* Abundant extraterrestrial amino acids in the primitive CM carbonaceous chondrite Asuka 12236. Meteorit Planet Sci 2020;55(9):1979–2006; doi: 10.1111/maps.13560

[B19] Greenwood NN, Earnshaw A. Silica and silicic acids. Chemistry of the Elements, 2nd edition. Elsevier: Butterworth-Heinemann, 1997; pp. 342–366.

[B20] Guélin M, Cernicharo J. Organic molecules in interstellar space: Latest advances. Front Astron Space Sci 2022;9:787567; doi: 10.3389/fspas.2022.787567

[B21] Hattori S, Kiriyama-Tanaka T, Kusubata M, *et al.* Preservation of collagen in the soft tissues of frozen mammoths. PLoS One 2021;16(10):e0258699. 34714842 10.1371/journal.pone.0258699 PMC8555803

[B22] Heck PR, Greer J, Kööp L, *et al.* Lifetimes of interstellar dust from cosmic ray exposure ages of presolar silicon carbide. Proc Natl Acad Sci U S A 2020;117(4):1884–1889; doi: 10.1073/pnas.1904573117 31932423 PMC6995017

[B23] Hirao I, Kimoto M, Mitsui T, *et al.* An unnatural hydrophobic base pair system: Site-specific incorporation of nucleotide analogs into DNA and RNA. Nat Methods 2006;3(9):729–735; doi: 10.1038/nmeth915 16929319

[B24] Horowitz NH, Hobby GL, Hubbard JS. Viking on Mars: The carbon assimilation experiments. J Geophys Res 1977;82(28):4659–4662; doi: 10.1029/JS082i028p04659

[B25] Kawaguchi Y, Shibuya M, Kinoshita I, *et al.* DNA damage and survival time course of deinococcal cell pellets during 3 years of exposure to outer space. Front Microbiol 2020;11:2050. 32983036 10.3389/fmicb.2020.02050 PMC7479814

[B26] Kissel J, Krueger F. The organic component in dust from comet Halley as measured by the PUMA mass spectrometer on board Vega 1. Nature 1987;326(6115):755–760.

[B27] Koga T, Naraoka H. A new family of extraterrestrial amino acids in the Murchison meteorite. Sci Rep 2017;7(1):636; doi: 10.1038/s41598-017-00693-9 28377577 PMC5428853

[B28] Kvenvolden K, Lawless J, Pering K, *et al.* Evidence for extraterrestrial amino-acids and hydrocarbons in the Murchison meteorite. Nature 1970;228(5275):923–926. 5482102 10.1038/228923a0

[B29] Lee H, Jeon JH, Lim JC, *et al.* Peptide nucleic acid synthesis by novel amide formation. Org Lett 2007;9(17):3291–3293; doi: 10.1021/ol071215h 17661472

[B30] Lowenstein TK, Schubert BA, Timofeeff MN. Microbial communities in fluid inclusions and long-term survival in halite. GSA Today 2011;21(1):4–9.

[B31] Margulis L, Mazur P, Barghoorn ES, *et al.* The Viking Mission: Implications for life on Mars. J Mol Evol 1979;14(1):223–232. 522154 10.1007/BF01732380

[B32] Marshall SM, Mathis C, Carrick E, *et al.* Identifying molecules as biosignatures with assembly theory and mass spectrometry. Nat Commun 2021;12(1):3033; doi: 10.1038/s41467-021-23258-x 34031398 PMC8144626

[B33] Martin W, Russell MJ. On the origins of cells: A hypothesis for the evolutionary transitions from abiotic geochemistry to chemoautotrophic prokaryotes, and from prokaryotes to nucleated cells. Philos Trans R Soc Lond B Biol Sci 2003;358(1429):59–85; doi: 10.1098/rstb.2002.1183 12594918 PMC1693102

[B34] Martins Z, Botta O, Fogel ML, *et al.* Extraterrestrial nucleobases in the Murchison meteorite. Earth Planet Sci Lett 2008;270(1–2):130–136.

[B35] Mitsui T, Kitamura A, Kimoto M, *et al.* An unnatural hydrophobic base pair with shape complementarity between pyrrole-2-carbaldehyde and 9-methylimidazo[(4,5)-b]pyridine. J Am Chem Soc 2003;125(18):5298–5307; doi: 10.1021/ja028806h 12720441

[B36] Moretto H-H, Schulze M, Wagner G. Silicones. In: Ullmann's Encyclopedia of Industrial Chemistry. Wiley-VCH Verlag GmbH & Co KGaA: Weinheim, 2000; pp. 675–712.

[B37] Nakamura E, Kobayashi K, Tanaka R, *et al.* On the origin and evolution of the asteroid Ryugu: A comprehensive geochemical perspective. Proc Japan Acad Ser B 2022;98(6):227–282; doi: 10.2183/pjab.98.015 35691845 PMC9246647

[B38] Oyama VI, Berdahl BJ. The Viking Gas Exchange Experiment results from Chryse and Utopia surface samples. J Geophys Res 1977;82(28):4669–4676; doi: 10.1029/JS082i028p04669

[B39] Pham VH, Kim J. Cultivation of unculturable soil bacteria. Trends Biotechnol 2012;30(9):475–484. 22770837 10.1016/j.tibtech.2012.05.007

[B40] San Antonio JD, Schweitzer MH, Jensen ST, *et al.* Dinosaur peptides suggest mechanisms of protein survival. PLoS One 2011;6(6):e20381; doi: 10.1371/journal.pone.0020381 21687667 PMC3110760

[B41] Schmitt-Kopplin P, Gabelica Z, Gougeon RD, *et al.* High molecular diversity of extraterrestrial organic matter in Murchison meteorite revealed 40 years after its fall. Proc Natl Acad Sci U S A 2010;107(7):2763–2768; doi: 10.1073/pnas.0912157107 20160129 PMC2840304

[B42] Schopf JW, Kitajima K, Spicuzza MJ, *et al.* SIMS analyses of the oldest known assemblage of microfossils document their taxon-correlated carbon isotope compositions. Proc Natl Acad Sci U S A 2018;115(1):53–58; doi: 10.1073/pnas.1718063115 29255053 PMC5776830

[B43] Tarter JC. Astrobiology and SETI. New Astron Rev 2004;48(11–12):1543–1549.

[B44] Tedeschi R, De Paoli P. Collection and preservation of frozen microorganisms. In: Methods in Biobanking. (Dillner J, ed.) Humana Press: Totowa, NJ, 2011; pp. 313–326. 10.1007/978-1-59745-423-0_18 20949399

[B45] van der Valk T, Pečnerová P, Díez-del-Molino D, *et al.* Million-year-old DNA sheds light on the genomic history of mammoths. Nature 2021;591(7849):265–269; doi: 10.1038/s41586-021-03224-9 33597750 PMC7116897

[B46] Voet D, Voet JG. Biochemistry, 4th edition. John Wiley & Sons, Inc.: Hoboken, NJ; 2010.

[B47] Zherebker A, Kostyukevich Y, Volkov DS, *et al.* Speciation of organosulfur compounds in carbonaceous chondrites. Sci Rep 2021;11(1):7410; doi: 10.1038/s41598-021-86576-6 33795703 PMC8016918

